# Detecting cord blood cell type-specific epigenetic associations with gestational diabetes mellitus and early childhood growth

**DOI:** 10.1186/s13148-021-01114-5

**Published:** 2021-06-26

**Authors:** Tianyuan Lu, Andres Cardenas, Patrice Perron, Marie-France Hivert, Luigi Bouchard, Celia M. T. Greenwood

**Affiliations:** 1 Lady Davis Institute for Medical Research, Jewish General Hospital, 3755 Chemin de La Côte-Sainte-Catherine, Montréal, QC H3T 1E2 Canada; 2 Quantitative Life Sciences Program, McGill University, Montréal, QC Canada; 3 Division of Environmental Health Sciences, School of Public Health and Center for Computational Biology, University of California, Berkeley, CA USA; 4 Department of Medicine, Université de Sherbrooke, Sherbrooke, QC Canada; 5 Centre de Recherche du Centre Hospitalier, Universitaire de Sherbrooke, Sherbrooke, QC Canada; 6 Diabetes Unit, Massachusetts General Hospital, Boston, MA USA; 7 Department of Population Medicine, Harvard Pilgrim Health Care Institute, Harvard Medical School, Boston, MA USA; 8 Department of Biochemistry and Functional Genomics, Université de Sherbrooke, Sherbrooke, QC Canada; 9 Department of Medical Biology, Centre Intégré Universitaire de Santé et de Services Sociaux Saguenay-Lac-Saint-Jean - Hôpital Universitaire de Chicoutimi, Saguenay, QC Canada; 10 Department of Epidemiology, Biostatistics and Occupational Health, McGill University, Montréal, QC Canada; 11 Department of Human Genetics, McGill University, Montréal, QC Canada; 12 Gerald Bronfman Department of Oncology, McGill University, Montréal, QC Canada

**Keywords:** Epigenome-wide association study, DNA methylation, Cell type specificity, Gestational diabetes mellitus, Early childhood growth

## Abstract

**Background:**

Epigenome-wide association studies (EWAS) have provided opportunities to understand the role of epigenetic mechanisms in development and pathophysiology of many chronic diseases. However, an important limitation of conventional EWAS is that profiles of epigenetic variability are often obtained in samples of mixed cell types. Here, we aim to assess whether changes in cord blood DNA methylation (DNAm) associated with gestational diabetes mellitus (GDM) exposure and early childhood growth markers occur in a cell type-specific manner.

**Results:**

We analyzed 275 cord blood samples collected at delivery from a prospective pre-birth cohort with genome-wide DNAm profiled by the *Illumina MethylationEPIC* array. We estimated proportions of seven common cell types in each sample using a cord blood-specific DNAm reference panel. Leveraging a recently developed approach named CellDMC, we performed cell type-specific EWAS to identify CpG loci significantly associated with GDM, or 3-year-old body mass index (BMI) z-score. A total of 1410 CpG loci displayed significant cell type-specific differences in methylation level between 23 GDM cases and 252 controls with a false discovery rate < 0.05. Gene Ontology enrichment analysis indicated that LDL transportation emerged from CpG specifically identified from B-cells DNAm analyses and the mitogen-activated protein kinase pathway emerged from CpG specifically identified from natural killer cells DNAm analyses. In addition, we identified four and six loci associated with 3-year-old BMI z-score that were specific to CD8+ T-cells and monocytes, respectively. By performing genome-wide permutation tests, we validated that most of our detected signals had low false positive rates.

**Conclusion:**

Compared to conventional EWAS adjusting for the effects of cell type heterogeneity, the proposed approach based on cell type-specific EWAS could provide additional biologically meaningful associations between CpG methylation, prenatal maternal GDM or 3-year-old BMI. With careful validation, these findings may provide new insights into the pathogenesis, programming, and consequences of related childhood metabolic dysregulation. Therefore, we propose that cell type-specific analyses are worth cautious explorations.

**Supplementary Information:**

The online version contains supplementary material available at 10.1186/s13148-021-01114-5.

## Background

With the advent of technologies such as microarrays, profiling genome-wide DNA methylation (DNAm) and conducting epigenome-wide association studies (EWAS) have become common approaches to explore epigenetic mechanisms underpinning complex traits and biological processes [1]. Biological samples collected to quantify DNAm, e.g. blood samples and other bulk tissues, are usually mixtures of different cell types. It has been recognized that cellular heterogeneity may strongly confound EWAS, because, in addition to cell type compositions possibly being associated with the phenotype of interest [2–4], DNAm also exhibits distinctive and substantial cell type-specific patterns in both healthy and diseased individuals [3]. Though it is desirable to separate and analyze different cells respectively by fluorescence-activated cell sorting (FACS) [5] or single-cell methylome sequencing [6, 7], such methods may not be easily generalizable to large epidemiological cohorts for complex traits due to potentially high cost and indispensable facilities.

Various statistical approaches have been proposed to infer cell type composition in samples and correct for cell type heterogeneity in EWAS [8–12]. However, many of these approaches consider that the observed or estimated cell type proportions may be associated with the phenotype, but not with differences in DNAm (at most of the measured positions) [8]. Therefore, these approaches to adjust for confounding by in-sample cell type proportions may have low power for identifying differentially methylated loci when such differences, which might not be evident, are specific to a subset of cell types, or demonstrate opposing directions in different cell types [4]. Recently, a new algorithm called CellDMC was developed and validated by Zheng et al. [4] to enable detection of cell type-specific differential DNA methylation by identifying interactions between the phenotype and cell type proportions in samples. The benefits of this approach in human samples containing cell type mixtures may worth extensive exploration.

Gestational diabetes mellitus (GDM) is a complex condition that has been associated with various adverse impacts on the development and growth of the offspring [13, 14]. Increasing evidence suggests crucial epigenetic mechanisms may be implicated in the varying consequences of GDM [15–17]. For instance, two regions, one in the autism spectrum disorder-related gene *OR2L13* and the other in the metabolic enzyme gene *CYP2E1*, had lower DNAm levels in cord blood of newborns exposed to GDM compared to controls [13]. Placental DNAm profiles of adipokines genes (e.g. *LEP* and *ADIPOQ*) and inflammatory genes (e.g. *PDE4B*) were also found to be associated with exposure to maternal hyperglycemia [18, 19] and maternal glycemia response [20], respectively. Nevertheless, it is still poorly understood whether such epigenetic variability is cell type-specific.

Therefore, to better survey the epigenetic mechanisms associated with gestational diabetes, we implemented CellDMC [4] for detecting cell type-specific DNAm in EWAS on GDM, leveraging cord blood samples collected from 275 mother–child pairs in the Genetics of Glucose Regulation in Gestation and Growth (Gen3G) cohort [21]. Additionally, we attempted to identify cord blood cell type-specific DNAm associated with 3-year-old body mass index (BMI) z-scores, an important marker of fetal and early childhood growth associated with later-life health outcomes [22]. We assessed the benefits of employing cell-type specific analyses (CellDMC) through comparison with a conventional EWAS framework and an alternative interaction detection framework.

## Methods

### Study cohort

The Gen3G cohort [21] is a prospective pre-birth cohort that was established between January 2010 and June 2013 at the Centre Hospitalier Universitaire de Sherbrooke, in Sherbrooke, Canada. Expecting mothers aged ≥ 18 years with a singleton pregnancy, who did not have pre-pregnancy diabetes mellitus as determined by medical history and screening were eligible for enrollment. Data and samples were collected at three time points during pregnancy: the first research visit between 5 and 16 weeks of gestation, the second visit between 24 and 30 weeks of gestation, and at delivery [21]. At the first trimester, trained research staff measured maternal height and weight using standardized protocols. Participating women reported age, current smoking status, and parity. Women subsequently underwent GDM screening during the second trimester of pregnancy using a 75 g oral glucose tolerance test (OGTT), and were identified as having GDM if one of the following criteria was fulfilled: (1) fasting glucose ≥ 5.1 mmol/L, (2) 1-h OGTT ≥ 10 mmol/L, or (3) 2-h OGTT ≥ 8.5 mmol/L, following the American Diabetes Association/International Association of Diabetes and Pregnancy Study Group (ADA/IADPSG) diagnostic criteria [23]. These women who were diagnosed with GDM received treatment according to 2008 Canadian Diabetes Association guidelines [24] adopted at that time at the institution.

For the current study, we analyzed 275 mother–child pairs of European ancestry with delivery that occurred ≥ 37 weeks of gestation, and had measured weight and height of children upon a return visit at the age of 3 when the offspring were aged 40.5 ± 3.0 months [25]. Weight was measured with a calibrated electronic scale with bare feet in light clothing. Height was measured with a wall stadiometer without shoes. Body mass index (BMI) was computed as z-scores using the World Health Organization (WHO) growth chart reference for boys and girls. Data from all 275 samples were used for DNAm-GDM association tests. Three children did not have age 3 BMI measures, thus only 272 samples were included in DNAm-age 3 BMI z-score association tests.

### Cord blood sample collection and measurement of DNAm

We collected whole blood samples of cord blood within 30 min after delivery by research staff, as described previously [21]. Samples were then aliquoted (300–500 µL/aliquot) and stored at − 80 °C. Double strand DNA concentration was assessed using Quant-iT™ PicoGreen™ dsDNA Assay Kit (Qiagen, USA) after DNA extraction using the All Prep DNA/RNA/Protein Mini Kit (Qiagen, USA). Methylation levels at > 850,000 CpG loci were quantified using the Infinium MethylationEPIC BeadChip after sodium-bisulfite conversion [21]. Preprocessing was preformed using the *minfi* R package [26]. We removed non-CpG probes, probes that were annotated to sex chromosomes, probes that showed non-significant detection (*p* value > 0.05) in ≥ 5% of the samples, probes with a single nucleotide polymorphism (SNP) with a minor allele frequency ≥ 5% at the target CpG or at the single base extension, as well as cross-reactive probes reported in [27]. We used the ComBat function in the *sva* R package to account for batch effects [28]. We retained a total of 790,563 high quality probes for statistical analyses.

### Estimation of cell type proportions

Cell type proportions for B-cells, CD4+ T-cells, CD8+ T-cells, granulocytes, monocytes, natural killer cells and nucleated red blood cells in each sample were estimated using the *minfi* R package with a cord blood-specific DNAm reference panel including 700 CpG loci that are differentially methylated between these seven cell types [29]. The accuracy of this reference panel has been previously verified in our study cohort [30]. Estimated cell type proportions were normalized such that they added up to 100% in each sample:

$${f}_{k,i}=\frac{{f}_{k,i,raw}}{\sum _{k=1}^{K}{f}_{k,i,raw}}$$

where ${f}_{k,i,raw}$ and ${f}_{k,i}$ represent the raw estimated cell type proportion and the normalized cell type proportion for the $k$-th cell type (of $K=7$ in total) of the $i$-th sample, respectively.

### Detecting cell type-specific differential methylation

We performed two epigenome-wide analyses, looking for interactions between cell type proportions and two variables: (1) GDM, and (2) 3-year-old BMI z-score. Per-locus association was tested separately for each of the phenotype variables, using the CellDMC framework proposed in Zheng et al. [4]:

1 $${x}_{c}={\sum }_{k}{f}_{k}{\mu }_{kc}+{\sum }_{k}{f}_{k}y{\beta }_{kc}+z\rho +e$$

Here, ${x}_{c}$ represents methylation M-value (logit-transformed methylation proportion) at probe $c$, ${f}_{k}$ represents cell type proportion of cell type $k$, $y$ and $z$ are the phenotype of interest and the additional covariates, respectively, and $e$ is random error. The estimated parameters are the cell type-specific means ${\mu }_{kc}$, the covariate effects $\rho$, and interaction terms ${\beta }_{kc}$, with the latter being of most interest in this study. For all analyses, we adjusted for maternal age, smoking status in early pregnancy (being a current smoker or not), parity, gestational age at birth, and child sex in the covariates, $z$.

In this framework, a significant interaction between the phenotype (*y*) and the corresponding cell type proportion will be observed if levels of methylation are correlated with $y$ in different ways across the cell types. This interaction effect may be significant even if the main effects of cell type proportion and the phenotype (*y*) are not significant. Also, since cell type proportions sum up to 100%, no intercept was included.

Notably, we used M-value in model (1) since it has been shown to be more statistically valid for linear model-based differential analysis of methylation levels than the $\beta$-value (methylation proportion) [31]. However, for a more intuitive biological interpretation, we also repeated the analyses using $\beta$-values. For DNAm-GDM association tests, the coefficient for the interaction effect ${\beta }_{kc}$ based on $\beta$-value could be interpreted as change in methylation proportion in cell type $k$ among individuals exposed to GDM compared to those not exposed to GDM, at probe $c$; For DNAm-age 3 BMI z-score association tests, ${\beta }_{kc}$ could be interpreted as change in methylation proportion in cell type $k$ associated with one unit increase in the BMI z-score, at probe $c$. Conclusions regarding statistical significance were drawn from the M-value analyses, but magnitudes of effect were explored on the $\beta$-value scale.

We compared results obtained using this framework to those by performing standard EWAS, testing for association between each probe and the phenotype y, while adjusting for the effects of the same covariates as well as the estimated cell type proportions, but not including the interactions.

### Alternative approaches for detection of interaction effects

The model presented in Eq. (1) is set up in a way that is statistically non-standard, because the phenotype, $y$, is on the independent-variable side of the model. We followed Zheng et al. [4] by using this model setup. Nevertheless, for BMI at age 3, we performed additional analysis with the equation:

2 $$y={\sum }_{k}{f}_{k}{\nu }_{k}^{*}+{\sum }_{k}{f}_{k}{x}_{c}{\gamma }_{kc}^{*}+{z\rho }^{*}+{e}^{*}$$

Here, different from the previous model (1), ${\nu }_{k}^{*}$ depicts the effect of cell type $k$ on the phenotype, while ${\gamma }_{kc}^{*}$ represents the effect of interaction between DNAm in cell type $c$ and the $k$-th cell type on the phenotype. We did not use Eq. (2) for the GDM phenotype since this was measured, in the mothers, prior to collection of the cord blood at birth. This model differs from Eq. (1) in that it assumes that the associations between cell type proportions and the phenotype $y$ vary depending on the probe-of-interest’s (logit-transformed) methylation level, whereas the former assumes that $y$ modulates the association between cell type proportions and the probe-of-interest’s (logit-transformed) methylation level.

### Verification of genome-wide significance

One important consideration is whether the results from our analyses are likely to have occurred by chance. Given the number of tests being performed—genome-wide interaction tests with each of seven cell types—robust adjustments for multiple testing are necessary. We therefore performed permutation analyses to look at genome-wide significance for these interaction tests. Designing appropriate permutation tests for interactions is challenging [32]. We followed the recommendations of Buzkova et al. [33, 34] by first fitting a model without any interaction terms, and calculating residuals $R={x}_{c}-{\widehat{x}}_{c}$ for each probe. We then permuted the residuals across the samples while ensuring the order of re-assignment was consistent for all probes and computed the test statistics for the interaction terms by refitting Eq. (1) using the permuted residuals instead of the original methylation levels. We repeated this procedure 100 times for each phenotype and obtained epigenome-wide distributions of *p* values. This approach retains the correlation across different probes and provides genome-wide null distributions of the *p* values.

### Functional annotation

For each cell type, we identified target genes if any cell type-specific differentially methylated CpG locus was located in a genic region, an upstream regulatory region (5′ untranslated region or up to 1,500 base-pairs upstream of the transcription start site), or a downstream regulatory region (3′ untranslated region). Gene Ontology (GO)-enrichment analysis was performed for these genes, for each cell type separately, using the enrichGO function in the *clusterProfiler* R package [35]. All known ontologies for biological processes, molecular functions and cellular components were included for enrichment analysis. Significantly enriched GO terms were defined as having a false discovery rate (FDR; *p* value adjusted for multiple testing by the Benjamini–Hochberg method) < 0.05.

## Results

### Cohort characteristics

Demographic and clinical characteristics of the 275 mother–child pairs used in this study are summarized in Table 1. At enrollment, the mean (SD) age was 28.5 (4.2) years and mean (SD) BMI was 25.5 (5.7) kg/m^2^. All mothers were Caucasian and 132 (48.0%) were primiparous. Twenty-three (8.4%) mothers had GDM. Nine of these GDM mothers were treated with insulin and 14 received dietary interventions. Characteristics of the mothers and the children were largely consistent between those exposed to GDM and those not exposed to GDM, except that mothers with GDM were more likely to be current smokers (Additional file 1: Tables S1 and S2). At delivery, newborns had a mean (SD) gestational age of 39.5 (1.0) weeks and birthweight of 3.4 (0.4) kg, and 150 (54.5%) were male. At the 3-year-old visit, the children had a mean (SD) BMI of 16.2 (1.6) kg/m^2^. Most estimated cell type proportions in the samples were not significantly associated with the phenotypes of interest, except that the estimated monocyte proportion was weakly associated with Fenton’s birthweight z-score [36] (Pearson correlation *r* = 0.17, FDR = 0.03) and age 3 BMI z-score (*r* = 0.16, FDR = 0.04). (Additional file 7: Supplementary Figure S1).

**Table 1:** Demographic and clinical characteristics of 275 Gen3G mother–child pairs included in this study

	Mean (SD)/*N* (%)
Mother	
Age (year)	28.5 (4.2)
Height (cm)	164.8 (6.4)
Weight (kg)	69.3 (15.6)
Body mass index (BMI; kg/m^2^)	25.5 (5.7)
Parity	
Being primiparous	132 (48.0)
Gestational diabetes mellitus^†^	23 (8.4)
Insulin therapy	9 (3.3)
Dietary intervention	14 (5.1)
Smoking (at 1^st^ trimester)	
Currently smoking	21 (7.6)
Child	
Gestational age at birth (week)	39.5 (1.0)
Male	150 (54.5)
Birthweight (kg)	3.4 (0.4)
Height (cm) at age 3^*^	96.9 (4.4)
Weight (kg) at age 3	15.2 (1.9)
BMI (kg/m^2^) at age 3^*^	16.2 (1.6)
Estimated cord blood cell type proportions (%)	
B-cell	9.5 (3.0)
CD4+ T-cell	15.8 (5.3)
CD8+ T-cell	12.6 (3.4)
Granulocyte	39.9 (9.2)
Monocyte	9.0 (2.6)
Natural killer cell	2.0 (2.6)
Nucleated red blood cell	11.2 (5.9)

^*^Three (1.1%) children had missing data and were not included in DNAm-age 3 BMI z-score association tests

^†^Demographic and clinical characteristics with respect to maternal gestational diabetes mellitus are presented in Additional file 1: Supplementary Tables S1 and S2

### Cell type-specific CpG methylation is associated with GDM exposure

**Fig. 1:**
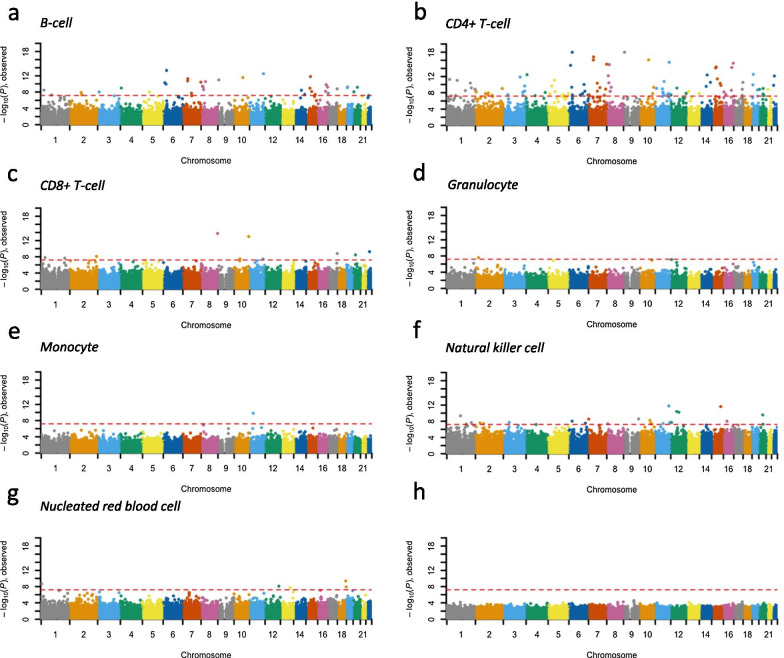
Manhattan plots summarizing epigenome-wide CpG methylation associated with gestational diabetes mellitus. (**a**–**g**) Cell type-specific differentially methylated CpG loci are indicated for seven cell types. Results obtained from standard EWAS adjusting for estimated cell type proportions are summarized in (**h**). CpG loci are aligned on the x-axis according to genomic coordinate and are colored by chromosome. The y-axis represents − log_10_ (*p* value). Red dashed lines denote Bonferroni-corrected genome-wide significance threshold (*p* value < 6.3 $\times$ 10^–8^)

We report here significant epigenome-wide findings for association tests using the model of Eq. (1) above across all autosomal CpGs, determined by an FDR threshold (FDR < 0.05). Detailed results are summarized in Additional file 2: Supplementary Tables S3. In total, 1410 CpG loci were found to be significantly associated with GDM in a cell type-specific manner (FDR < 0.05; Fig. 1a–g; Additional file 2: Supplementary Table S3), 1174 of which were located in genic regions or potential upstream or downstream regulatory regions, involving 1282 genes. Of these 1410 CpG loci, in the corresponding cell type, 216 were estimated to be completely methylated among individuals exposed to GDM but unmethylated among those not exposed to GDM (${\beta }_{kc}$ estimated to be 1 in model (1) based on $\beta$-value), while 159 were estimated to be unmethylated among individuals not exposed to GDM but completely methylated among those exposed to GDM (${\beta }_{kc}$ estimated to be −1 in model (1) based on $\beta$-value). Furthermore, 143 loci reached a more conservative Bonferroni-corrected genome-wide significance (*p* value < 0.05/790,563 = 6.3 $\times$ 10^–8^). Of these 143 loci, 108 had effects unique to one cell type while 35 had effects in two different cell types (Additional file 2: Supplementary Table S3). In contrast, no apparent association was observed in a cell type proportion-adjusted standard EWAS (Fig. 1h). Cell type-specific GO-enrichment analyses (Methods) on genes harboring these significantly methylated CpG loci (FDR < 0.05) indicated that some diabetes mellitus-relevant pathways were probably involved in a cell type-specific manner, such as LDL transportation emerging from GDM-hypermethylated gene body CpG loci of *LDLR*, *SCARF1* and *SORL1*, specifically identified from B-cells DNAm analyses, and the mitogen-activated protein kinase pathway emerging from CpG loci involving 18 genes, including GDM-hypermethylated upstream regulatory region CpG loci of *MAPK11*, *MAP3K10*, and *MAP3K12*, specifically identified from natural killer cells DNAm analyses (Additional file 3: Supplementary Table S4).

### Cell type-specific CpG methylation possibly associated with early childhood growth

**Fig. 2:**
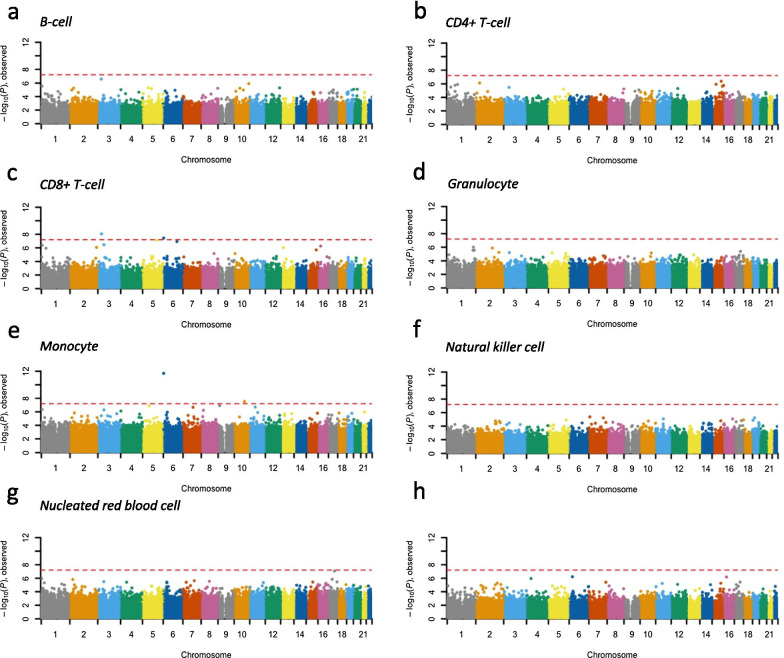
Manhattan plots summarizing epigenome-wide CpG methylation associated with 3-year-old BMI z-score. (**a**–**g**) Cell type-specific differentially methylated CpG loci are indicated for seven cell types. Results obtained from standard EWAS adjusting for estimated cell type proportions are summarized in (**h**). CpG loci are aligned on the x-axis according to genomic coordinate and are colored by chromosome. The y-axis represents − log_10_ (*p* value). Red dashed lines denote Bonferroni-corrected genome-wide significance threshold (*p* value < 6.3 $\times$ 10^–8^)

When we investigated the offspring 3-year-old BMI z-score, we observed four gene body CpG loci associated with 3-year-old BMI z-score in CD8 + T-cells (first two were significant after Bonferroni correction): cg02702424 (*TGFBR2*, $\beta$-value decreased 0.25 per unit increase in BMI z-score), cg12586150 (*SERPINB1*, $\beta$-value increased 0.30 per unit increase in BMI z-score), cg18813020 (*PRDM6*, $\beta$-value decreased 0.09 per unit increase in BMI z-score), and cg25821794 (*VGLL2*, $\beta$-value decreased 0.10 per unit increase in BMI z-score); as well as six loci associated with 3-year-old BMI in monocytes (first two were Bonferroni significant; Fig. 2a–g; Additional file 4: Supplementary Table S5): gene body locus cg12586150 (*SERPINB1*, $\beta$-value decreased 0.48 per unit increase in BMI z-score), upstream regulatory region locus cg00974033 (*BMPR1A*, $\beta$-value decreased 0.60 per unit increase in BMI z-score), gene body locus cg06166187 (*MPDZ*, $\beta$-value decreased 0.32 per unit increase in BMI z-score), gene body locus cg19418629 (*ANKRD55*, $\beta$-value decreased 0.34 per unit increase in BMI z-score), cg10950644 (intergenic region, $\beta$-value decreased 0.54 per unit increase in BMI z-score), and cg02213440 (intergenic region, $\beta$-value decreased 0.54 per unit increase in BMI z-score). Again, no significant association was identified using standard cell type proportion adjusted epigenome-wide association test for 3-year-old BMI z-score (Fig. 2h).

### Validation by permutation

**Fig. 3:**
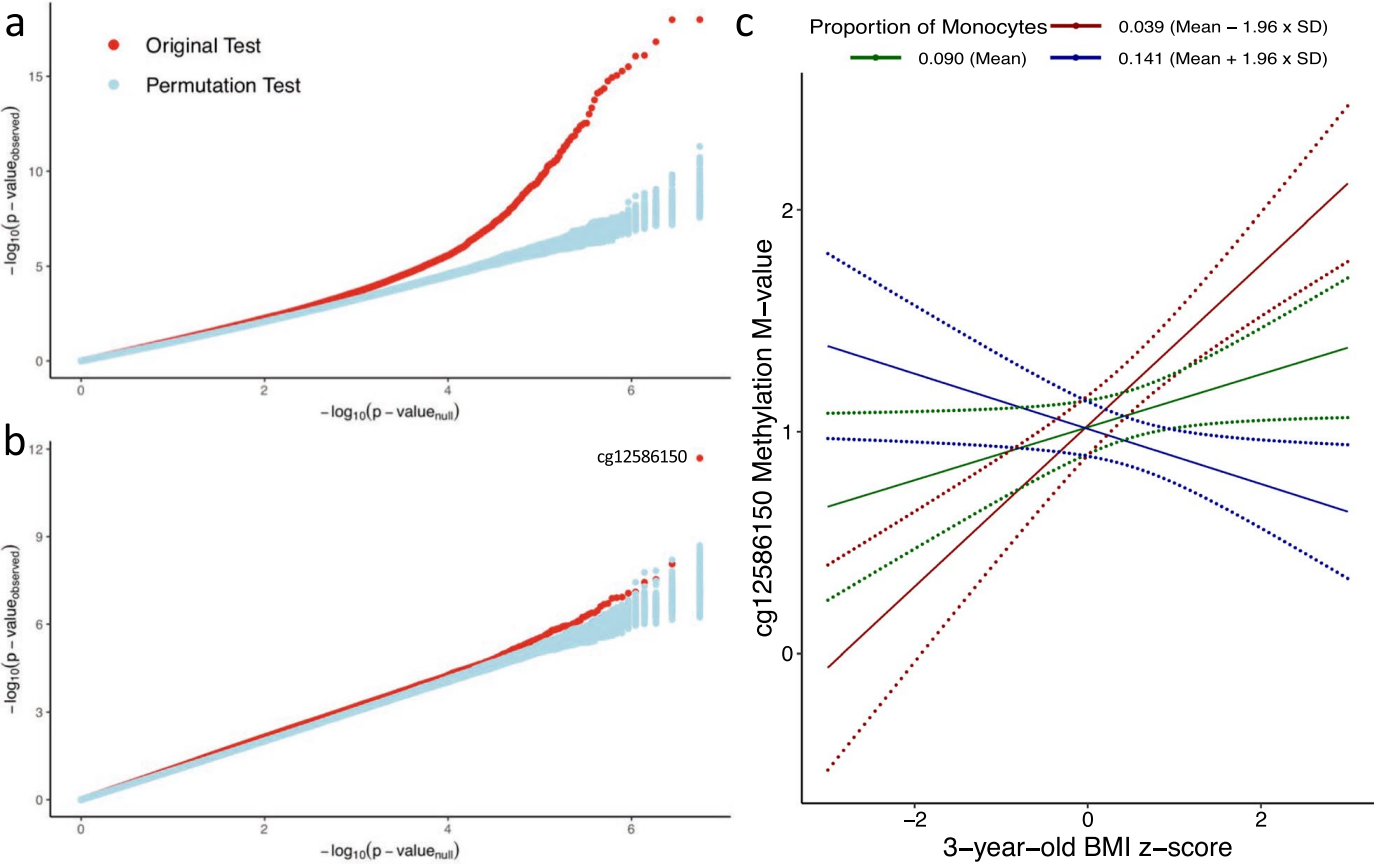
Quantile–Quantile plots of *p* values in epigenome-wide permutation tests. Ten permutations for association tests for (**a**) gestational diabetes mellitus and (**b**) 3-year-old BMI z-score were performed respectively. Distributions of *p* values obtained in these permutations are compared to those (red dots) obtained in the original analysis of cell type-specific differential methylation. All significant CpG loci associated with gestational diabetes mellitus (FDR < 0.05) reside on the right side of the curve after inflexion. (**c**) Association between 3-year-old BMI z-score and methylation level at cg12586150 in *SERPINB1*. Solid lines indicate predicted effects; Dotted lines delineate 95% confidence intervals. For visualization, predictions were based on median maternal age, non-smoker, no parity, median gestational age, and child being female. Cell type proportions of other six cell types were set to $\frac{1 - Proportion\ of\ Monocytes}{6}$

Figure 3a and b illustrate Quantile–Quantile (QQ)-plots of genome-wide interaction *p* values obtained from tests performed using the original data and in 100 permutations. We observed that many probes demonstrated evidence of interactions for GDM; and these interactions occurred in various cell types. In contrast, there were only a handful of probes where *p* values were smaller than chance for BMI at age 3, with one apparent outlier. This top probe was cg12586150 residing in the gene body of *SERPINB1*, with an outstanding monocyte-specific effect and suggestive CD8+ T-cell-specific effect. Figure 3c illustrates interaction at this locus. When the proportion of monocyte is low (e.g. 3.9%, corresponding to the lowest 5% of the population), a high 3-year-old BMI z-score is associated with a higher methylation level, whereas this association attenuates when the proportion of monocyte becomes higher, and eventually reverses, e.g. when the proportion of monocyte is over 14.1% (corresponding to the highest 5% of the population).

### Alternative interaction detection

When we used the model of Eq. (2) instead, we found 37 interaction effects between monocyte proportion and methylation level on age 3 BMI z-score (FDR < 0.05; Additional file 5:  Supplementary Table S6), among which only one probe, cg14571620 (*C12orf65*), reached Bonferroni-corrected genome-wide significance. Two of these 37 probes, cg10950644 (intergenic region) and cg06166187 (*MPDZ*), also demonstrated monocyte-specific effects in Eq. (1), suggesting potentially complicated associations at these loci. Additionally, we identified one interaction effect between methylation level and granulocyte proportion reaching Bonferroni-corrected genome-wide significance at cg27648960 in an uncharacterized gene *LOC100130093*. Using Eq. (2), we did not find association with the *SERPINB1* probe that stood out in Fig. 3c.

## Discussion

In this study, leveraging a recently developed method to detect differential methylation in a cell type-specific manner, we have performed correlative analyses to investigate the associations between cord blood CpG methylation, GDM and child BMI at age 3 in a cohort of moderate sample size (*N* = 275).

Compared to a conventional EWAS framework, our study yielded substantially more insights by explicitly modelling cell type-specific effects, though having not been validated externally. Our most interesting findings arise for GDM, and in this case the gestational diabetes status of the mother during the first trimester was ascertained prior to measuring the methylation levels at birth, facilitating interpretation of results. As expected, we identified cell type-specific differential DNAm in genes vital to carbohydrate and lipid metabolism, including *ADIPOR2* (gene body locus cg12568001, $\beta$-value decreased 0.68 among individuals exposed to GDM in CD4+ T-cell, and increased 0.89 in B-cell) [37], *GNAS* (upstream regulatory region locus cg03908391, unmethylated among individuals exposed to GDM but completely methylated among those not exposed to GDM in CD8+ T-cell) [38], *GRB10* (upstream regulatory region locus cg00228281, $\beta$-value increased 0.12 among individuals exposed to GDM in CD4+ T-cell) [39], etc. Moreover, we also identified differentially methylated genes known to be associated with maternal insulin sensitivity during pregnancy, such as *DLGAP2* (gene body loci cg20257821, cg02641770, and cg18540249, $\beta$-value decreased 0.24, 0.92 and 0.57 respectively among individuals exposed to GDM in CD8+ T-cell), *H19/MIR675* (gene body/upstream regulatory region locus cg16153294, $\beta$-value increased 0.46 among individuals exposed to GDM in natural killer cell), and *KCNQ1* (gene body locus cg06719391, completely methylated among individuals exposed to GDM but unmethylated among those not exposed to GDM in natural killer cell, and gene body locus cg21752270, $\beta$-value decreased 0.97 among individuals exposed to GDM in B-cell) [40]. Notably, existing studies suggest that many of these genes are subject to genomic imprinting, including in placenta [40, 41]. It has been proposed that the parental origin of imprinted regions in placenta may have a profound influence on nutrient transfer during pregnancy, in particular, contributing to the “maternal–fetal conflict” in regulating nutrient allocation [42]. Specifically, paternally imprinted genes (that express the maternal alleles) tend to prioritize maintenance of maternal resources, whereas maternally imprinted genes (that express the paternal alleles) may increase supply to the fetus [42, 43]. Given that the estimated cell type-specific methylation proportion changes were strong and may be of biological relevance, our findings imply that the underlying regulatory mechanisms might have cell type-specific activities which warrant future research.

Various diabetes-relevant pathways were identified in immune cells in enrichment analyses of the genes harboring cell type-specific DNAm significantly associated with GDM. Lipid dysfunction in diabetes has been widely characterized [44–46]. Previously, mononuclear cell surface expression of LDL receptor was found to decrease in type 2 diabetic patients [47]. Maternal total cholesterol level variations throughout pregnancy have been associated with placental DNAm in the *LDLR* and *LRP1* genes [48], where DNAm in *LRP1* may mediate the effect of changes in maternal blood lipid levels on cord blood leptin levels [48], a biomarker of adiposity. Here, we found that multiple lipid receptor-related pathways associated with lipid transportation and signaling might be influenced in a B-cell-specific manner, involving *LDLR* (cg16647139), *SORL1* (cg26556630), *SCARF1* (cg17028259), etc. In B-cells, these gene body CpG loci were estimated to be completely methylated among individuals exposed to GDM but were completely unmethylated among those not exposed to GDM. Natural killer cell-specific differential methylation was found to occur in *LRP1* (gene body locus cg20668447, $\beta$-value increased 0.23 among those exposed to GDM). Besides, pathways informative of other immune cell-specific functions also had strong enrichment of genes harboring differentially methylated CpGs, such as lytic functions of natural killer cells, involving mitogen-activated protein kinase activities and lysosome structures [49–51]. These identified targets did not overlap with an alternative framework in which cell type-specific effects are assumed to be dependent on the cell type proportions. While it is important to note that identification of differentially methylated CpG loci does not directly quantify changes in the amount of gene products, gene functions or pathway activities, particularly when DNAm in gene body may have a non-monotonic effect [52], we posit that these findings may implicate further investigations into the role of immune cell DNAm in GDM and its potential impacts on fetal development.

For 3-year-old BMI z-score, only one probe really stood out in our interaction analyses using the novel framework: a monocyte-specific association with cg12586150 in *SERPINB1*, which codes for the monocyte / neutrophil-derived elastase inhibitor and has been found associated with BMI z-score at age 5 in a genome-wide association study [53]. Plasma levels of serpinB1 have also been associated with insulin sensitivity in non-diabetic adults [54]. Therefore, further investigations with a focus on the role of specific cell types may be implicated, though these investigations should be undertaken knowing that the estimated monocyte proportion was itself moderately associated with age 3 BMI in our study.

We realize that in Eq. (1), methylation levels at specific probes are used as dependent variables, while simultaneously cell type proportions—estimated from the methylation levels in the same samples at hundreds of probes—are used as independent variables. A small set of only 700 probes was used for estimation of cell type convolution, and we posit that this is unlikely to have a substantial impact on our analysis. None of the significant probes we identified were among these 700 probes, and they displayed, at most, only moderate correlation with these reference probes (Additional file 6: Supplementary Table S7). Perhaps of more concern, if there are technical factors associated with genome-wide methylation levels that were not adequately removed during normalization, then there will be spurious correlations between the dependent and independent variables. Arguing against this hypothesis, our QQ-plots show little evidence of generalized inflation. Despite these reassurances, we recommend cautious interpretation of our results, particularly when these findings have not been validated in other cohort studies or experimentally.

In our modelling of these complex associations, the underlying assumptions should be carefully considered. First of all, linear models for methylation-phenotype associations, such as Eq. (1) but without the interactions, implicitly assume that the phenotype exerts a constant-sized effect on the logit-transformed methylation proportions (M-values), which corresponds to constant multipliers on the original proportion scale. In contrast, although the interaction models allow these multipliers to vary by cell type, there are still inherent assumptions on the relationship between the covariates and the mean methylation levels through the logit transformation. Furthermore, for a phenotype or covariate that exerts a strong influence on cell type proportions (e.g. an infection), spurious associations can be expected between probes known to have cell type-specific profiles and the proportions ${f}_{k}.$ This must be considered when speculating on potential interpretations of our results. Also, importantly, our study does not attempt to make statements about causal relationships which would need to be addressed through a formal causal modelling framework. For BMI z-score at age 3 in particular, the dependent variables, i.e. methylation levels, were profiled three years earlier than the independent variable, in Eq. (1), thereby creating an incompatible model if causal effect interpretations are desired. However, our study has provided an exploratory overview of epigenome-wide associations with gestational metabolism and with early childhood growth specific to seven cell types in cord blood. We anticipate future studies, including larger cohort studies with sufficient power to relax strong assumptions, and experimental studies on directly purified cell types by FACS [5] or single-cell sequencing [6], should be able to detect and validate more complicated non-homogeneous associations or causal effects.

## Conclusions

Gestational diabetes mellitus and early childhood growth may be associated with DNAm variations specific to certain cell types in cord blood. By implementing a novel discovery framework for interaction effects and genome-wide permutation tests, we demonstrate that these cell type-specific associations may be robustly identified. Such cell type-specific analyses are worth cautious explorations.

## Supplementary Information

**Additional file 1: Table S1**. Demographic and clinical characteristics of 23 Gen3G mother-child pairs with maternal gestational diabetes mellitus. **Table S2**. Demographic and clinical characteristics of 252 Gen3G mother-child pairs without maternal gestational diabetes mellitus.

**Additional file 2: Table S3**. Cell type-specific CpG methylation significantly associated with gestational diabetes mellitus.

**Additional file 3: Table S4**. Gene Ontology enrichment based on target genes harboring CpG with cell type-specific differential methylation associated with gestational diabetes mellitus.

**Additional file 4: Table S5**. Cell type-specific CpG methylation significantly associated with 3-year-old body mass index z-score.

**Additional file 5: Table S6**. Significant findings of interaction effects under the alternative interaction detection framework.

**Additional file 6: Table S7**. Pearson correlation between cell type-specific EWAS loci and reference loci for cell type deconvolution.

**Additional file 7.** Figure S1. Association between estimated cell type proportions and (a) gestational diabetes mellitus, (b) Fenton’s birthweight z-score and (c) 3-year-old BMI z-score. Pearson correlation (r) estimates are displayed. Bonferroni-corrected association *p* values were estimated using logistic regression for gestational diabetes mellitus or linear regression for z-scores, adjusted for maternal age, smoking status, parity, gestational age, and child sex.

## Data Availability

Data from the Gen3G cohort are available upon successful project application made to the Gen3G cohort committee. Full summary statistics of cell type-specific EWAS and all scripts for conducting computational analyses are available at https://figshare.com/articles/journal_contribution/Detecting_cord_blood_cell_type-specific_epigenetic_associations_with_gestational_diabetes_mellitus_and_early_childhood_growth/14562696.
