# Supplementary material for: Detecting cord blood cell type-specific epigenetic associations with gestational diabetes mellitus and early childhood growth
Source: Clin Epigenetics. 2021 Jun 26;13:131. doi: 10.1186/s13148-021-01114-5 (PMC8236204; doi:10.1186/s13148-021-01114-5)
Supplement: Supplementary file 1 — Additional file 1: Table S1. Demographic and clinical characteristics of 23 Gen3G mother-child pairs with maternal gestational diabetes mellitus. Table S2. Demographic and clinical characteristics of 252 Gen3G mother-child pairs without maternal gestational diabetes mellitus. [file 13148_2021_1114_MOESM1_ESM.docx]

**Table S1.** Demographic and clinical characteristics of 23 Gen3G mother-child pairs with maternal gestational diabetes mellitus.

|  | Mean (SD) / N (%) |
| --- | --- |
| Mother |  |
| Age (year) | 27.9 (4.5) |
| Height (cm) | 161.2 (6.2) |
| Weight (kg) | 68.0 (15.0) |
| Body Mass Index (BMI; kg/m^2^) | 26.1 (5.4) |
| Parity |  |
| Being primiparous | 11 (47.8) |
| Smoking (at 1^st^ trimester) |  |
| Currently smoking | 4 (17.4) |
|  |  |
| Child |  |
| Gestational age at birth (week) | 39.1 (0.8) |
| Male | 13 (56.5) |
| Birthweight (kg) | 3.5 (0.4) |
| Height (cm) at age 3 | 97.0 (3.0) |
| Weight (kg) at age 3 | 15.8 (3.1) |
| BMI (kg/m^2^) at age 3 | 16.8 (2.6) |
|  |  |
| Estimated cord blood cell type proportions (%) |  |
| B-cell | 8.8 (3.4) |
| CD4+ T-cell | 16.8 (6.3) |
| CD8+ T-cell | 11.3 (4.4) |
| Granulocyte | 40.6 (10.4) |
| Monocyte | 9.2 (3.0) |
| Natural killer cell | 1.2 (2.0) |
| Nucleated red blood cell | 12.0 (7.3) |

**Table S2.** Demographic and clinical characteristics of 252 Gen3G mother-child pairs without maternal gestational diabetes mellitus.

|  | Mean (SD) / N (%) |
| --- | --- |
| Mother |  |
| Age (year) | 28.5 (4.2) |
| Height (cm) | 165.2 (6.3) |
| Weight (kg) | 69.4 (16.0) |
| Body Mass Index (BMI; kg/m^2^) | 25.5 (5.8) |
| Parity |  |
| Being primiparous | 121 (48.0) |
| Smoking (at 1^st^ trimester) |  |
| Currently smoking | 17 (6.7) |
|  |  |
| Child |  |
| Gestational age at birth (week) | 39.5 (1.0) |
| Male | 137 (54.4) |
| Birthweight (kg) | 3.4 (0.3) |
| Height (cm) at age 3^*^ | 96.9 (4.5) |
| Weight (kg) at age 3 | 15.2 (1.7) |
| BMI (kg/m^2^) at age 3^*^ | 16.2 (1.5) |
|  |  |
| Estimated cord blood cell type proportions (%) |  |
| B-cell | 9.6 (3.1) |
| CD4+ T-cell | 15.7 (5.2) |
| CD8+ T-cell | 12.7 (3.3) |
| Granulocyte | 39.9 (9.1) |
| Monocyte | 9.0 (2.6) |
| Natural killer cell | 2.1 (2.6) |
| Nucleated red blood cell | 11.1 (5.8) |

^*^ Three children had missing data and were not included in DNAm-age 3 BMI z-score association tests.
