# Supplementary figures and images for: Detecting cord blood cell type-specific epigenetic associations with gestational diabetes mellitus and early childhood growth
Source: Clin Epigenetics. 2021 Jun 26;13:131. doi: 10.1186/s13148-021-01114-5 (PMC8236204; doi:10.1186/s13148-021-01114-5)

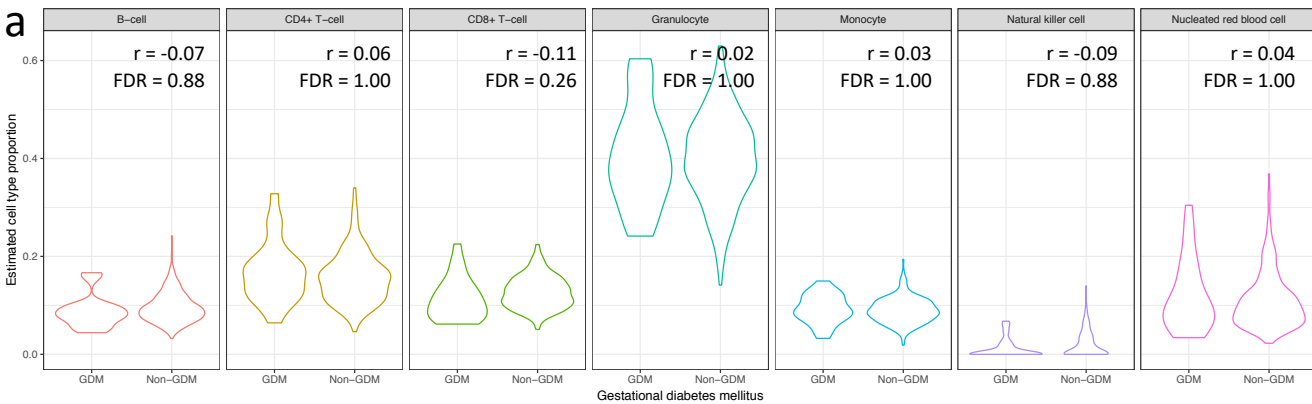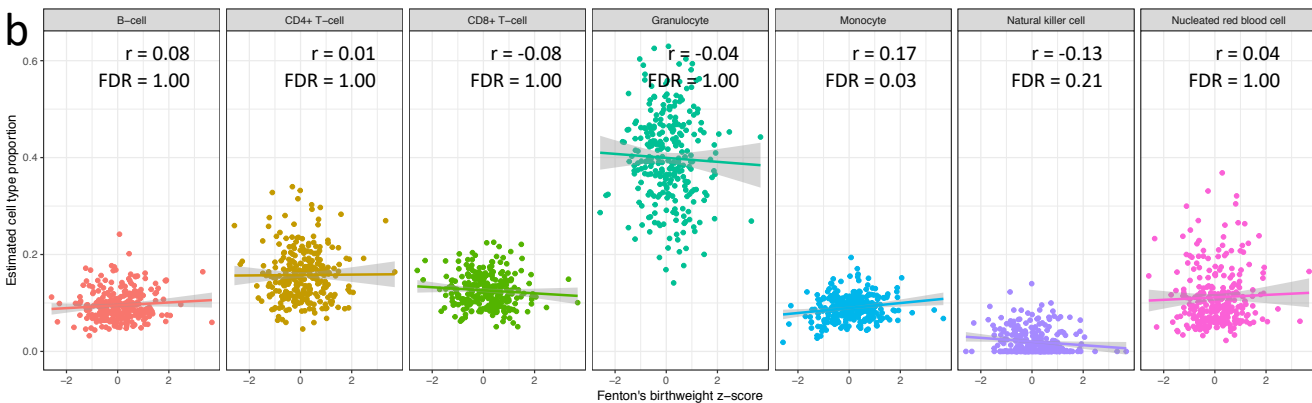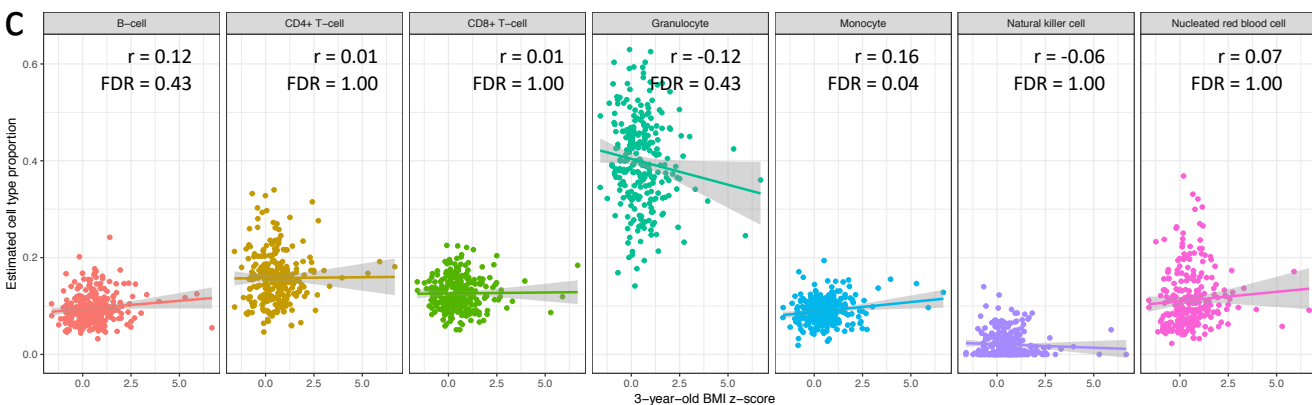

Supplement: Supplementary file 7 — Additional file 7. Figure S1. Association between estimated cell type proportions and (a) gestational diabetes mellitus, (b) Fenton’s birthweight z-score and (c) 3-year-old BMI z-score. Pearson correlation (r) estimates are displayed. Bonferroni-corrected association p values were estimated using logistic regression for gestational diabetes mellitus or linear regression for z-scores, adjusted for maternal age, smoking status, parity, gestational age, and child sex. [file 13148_2021_1114_MOESM7_ESM.pdf]
